# The American System of Medical Education*[In asserting in our last number the superiority of what we dignified with the name of the American System of Medical Education, we did not intend to claim for it perfection, or to be ranked even as very stringently conservative in regard to it. That it has its shortcomings, its sins of omission and commission, is very obvious. We are progressive in all our habits of thought, and acknowledge perfection in nothing of human origin. But, to repeat ourselves, we regard the system itself as satisfactory;—the manner in which it is carried out is another consideration; and as bearing upon these points, submit the above communication from the pen of a well-known lecturer and writer, whose views, while they do not altogether coincide with our own, are evidently those of an able and well-balanced mind. We publish the article upon its own merits.—Ed.]

**Published:** 1857-07

**Authors:** 


### ART. VIII.—*The American System of Medical Education.**

EXCELLENCE of plan and faultiness of detail are by no means incompatible. Some bridges are built, from the necessity of the case, of perishable material; yet so cunningly are they fitted together—without mortice or tenon—that any decayed piece may be taken out and replaced by something better. Like such a bridge, is the American System of Medical Teaching. It is a good bridge, it carries us safe over, but we must remove from it the rotten timbers, ere they give way and crush us in the fall.

It requires no uncommon shrewdness to point out the shaky supports in our construction; to provide proper substitutes may call for a wiser forethought and more careful deliberation, as to how and when our reform shall be put in operation.

It is consolatory to notice the fact, that the faults of our system are not inherent, but have their origin in some departure from the simplicity of the original idea. Thus, when the four months' course of lectures was established, it was supposed that the alternation of eight months of reading, with four of public instruction, would afford an agreeable variety to the student, and allow him an opportunity of reversing the ordinary mode of hibernation, by taking his full meal of mental aliment in the winter, and retiring to suck his paws in a quiet office during summer. In furtherance of this idea, it was ordained that a third course of lectures should be unattended by other expense than the matriculation and graduation fees; thus holding out a strong inducement to the tyro to sit at the feet of his Gamaliels three terms, instead of the obligatory two.

But here crept in a departure from the fathers, in the shape of sundry double-barrelled schools,—this happened before the days of revolvers—wherein two courses of lectures were given annually. In these institutions, the student was set up on the benches early in August, and regularly peppered with medical lore six hours daily, until the close of November, when he was turned loose to recover from his headache until March; then sub-

* [In asserting in our last number the superiority of what we dignified with the name of the American System of Medical Education, we did not intend to claim for it perfection, or to be ranked even as very stringently conservative in regard to it. That it has its shortcomings, its sins of omission and commission, is very obvious. We are progressive in all our habits of thought, and acknowledge perfection in nothing of human origin. But, to repeat ourselves, we regard the system itself as satisfactory;—the manner in which it is carried out is another consideration; and as bearing upon these points, submit the above communication from the pen of a well-known lecturer and writer, whose views, while they do not altogether coincide with our own, are evidently those of an able and well-balanced mind. We publish the article upon its own merits.—ED.]

jected to a repetition of the same old lectures from the same solemn professors, until the close of June, when, on some hot summer afternoon, he was discharged with a diploma, just in time to reap the benefits of dysentery and autumnal fevers.

This plan of instruction is still pursued in some rural institutions. Its chief merit is in the abundant opportunity it offers for cheating as to time, and the convenience it affords for students rejected at some respectable school; permitting them to forget their disgrace, and placing their fortunes in gentler hands, to graduate any how, at the end of four months from the time of their rejection. Aside from these incidental advantages, they hold out the inducements of cheapness, and it must not be forgotten that a systematic cramming for eight months in the year supposes remarkable powers of digestion on the part of the student. Another pleasant feature is, that it allows professors to earn more money with less work, and particularly benefits those peripatetic philosophers, who go about doing good and dividing the influence of their names in three or four different schools, where, not unfrequently, they teach three or four different branches.

Satire aside, this system of two terms annually, in which the same lectures are repeated, is hurtful and injurious to the cause of education, and a proper subject for the censures of the profession and the press.

Closely analogous to the double-barrelled colleges are the revolving professors, those men so surcharged with science that no less than three or four professorships, in different schools, can give vent to their erudition. The revolving professor is an animal *sui-generis.* Not unfrequently he teaches different departments in each of his schools; his brain is multilocular, and the different sections of it are closely packed with as many kinds of learning. This nomadic method of teaching is full of serious faults. The influence of the teacher is divided among the various schools in which he has chairs, so as to be nearly neutralized. He is only attached to a school as his pecuniary interest may dictate, and is ready to leave it at any time when it most needs his aid. Throwing aside these considerations of interest, however, it is evident that he who attempts to give more than one thorough course yearly, attempts too much, especially if he essays more than a single branch.

A scanty professional corps is another fault of some schools. In no form of effort is a division of labor more productive than in medical teaching, and without it the matter taught is bare, compendious, and lifeless, unassociated in the speaker's mind with any effort of his own, or any attempt to attain distinction in a subject of which he knows but little himself. The teacher who is compelled to embrace within his course a large range of subjects, must be a miracle of industry if he would undertake the condensation necessary to teach them well.

Of all the serious faults connected with our system, the worst is the attempt to dispense with clinical teaching. By this time the doctrine should be established, that no school should exist without the advantage of clinical instruction for its students. It seems absurd to argue this question, but it is only a very few years since our national association published in its proceedings an elaborate argument intended to prove that disease could be taught as well in the lecture-room as in the ward. The particular process of reasoning by which this marvellous result was reached does not now recur to me, except that it was proposed to confine the duty of the college to didactic instruction, leaving the student to his private instructor for bedside teaching. We will not stop to discuss such a proposition, for it is to me, and I trust to the readers of the "Review" sufficiently obvious that no school of medicine should be established or maintained where it has not the benefit of the admission of its class to some sufficient hospital. I am aware of the objections raised, of that tender humanity which repeats the old wives' story, that a hospital is designed as a safe refuge for the sick, and not as a theatre for medical display and experiment. I have seen all this and much more similar balderdash repeated during the past month, in a medical journal of considerable pretension to character, but I have no patience with it and no words to express my aversion to so narrow-minded a view.

I take the other ground. I assert that one great good to be derived from a hospital, a good sufficient in itself to induce its foundation, is the opportunity for observing disease collectively, and for pointing out to the student its various phases and conditions. I would go further: an obstetric clinic is not in itself objectionable. It *can* be made so offensive and vulgar as to shock the moral sense of the most obtuse, but it *will not* in the hands of the good men who make up the list of our professors of obstetrics. But I can imagine something worse than vulgarity,—a young graduate, without the vaguest knowledge of the practical manipulations of the art, or the etiquette of the lying-in room, in charge of a case of placenta prævia. I know of obstetric clinics where no sense of modesty, however fine, is disturbed, and which still afford to the advanced student abundant means of practical instruction.

I repeat, that where the means of clinical instruction are not available, no medical school should exist, and the attempt to maintain one is a quackery, if to claim to perform that which one cannot do, constitutes the true definition of quackery.

Thus far in my enumeration of evils, I have mentioned only those which are limited to a comparatively small number of schools. Many of our colleges are entirely free from them, and everywhere these evils seem to be on the decrease. But very few schools now hold two courses annually, and some of the older colleges have taken the bold position of refusing to graduate students who thus crowd all their public instruction into a single year. Most of the schools, also, have learned that the itinerant professors are not the most desirable of colleagues; and policy, if not the good of the profession, leads them to insist on single chairs. In others, too, may be noticed enlargement of the faculty, the separation of collateral from the practical branches, as in the creation of distinct chairs of pathology; and there are but few who now dare to take open ground against the necessity of clinical instruction. That any should do it, is a disgrace to American medicine.

The tendency, then, is toward reformation. But little talk is needed. It is in the power of the better schools to either kill out or reform the inferior order, just as the latter may be possessed of vitality. The noble competitive element in our system is already doing this. Let colleges which possess unusual advantages say so in their circulars, in any manly, straightforward way; let them use the great engine of modern business, the advertisement; let them consider teaching as a business which ought to pay if honorably conducted, and see to it that no absurd old fogyish notions of dignity interfere with their just claims to consideration. There are a dozen schools whose death is eminently desirable, and any honorable means which will hasten their final departure will do a great public good.

I would that I could stop here, and feel that I had touched on all the faults of our system; I would that those nobler schools which perpetuate the excellencies of the palmy past of American medicine, or those junior institutions ennobled by true science, by energy, and by faithful labor, were beyond and above the reach of the critic. In all of these there is so much that is grand and honest, so much self-sacrifice, so much true zeal for the honor of the profession, that I unwillingly touch on faults which they possess, and which call for remedy. Fortunately for us, these are not errors of the men of the present, but heir-looms of the past, come down to us from primitive days and engrafted in our system in its feeble infancy, when the wisest and the boldest of the fathers were compelled to submit to their inexorable surroundings. I can speak here in kindly tones, for I know that I address noble hearts, which only wait for the proper time and opportunity to beat with one common throb in any enterprise which promises good.

A reform, which will soon be loudly called for, and which is rapidly becoming inevitable, is an extension of collegiate instruction through the year; the distribution of the various branches into regular terms appropriated to them; and the diminution of the importance of the present winter course, by reducing its number of daily lectures to the capacity of the student.

I am aware that this is a broad and almost radical change, and that it involves within itself many questions of the gravest importance. Let me first sketch my ideal of a medical college, and then consider its advantages and the arguments to be brought against it.

1. The establishment of a collegiate year divided into two terms, one of four, and the other of five months. The first course to commence on the first of March, and continue until the first of July; to be followed by a vacation of two months during the hot season. The second course to begin on the first of September, and hold till the close of January.

2. The same professorships as are now maintained in our best schools.

3. During the year two lectures daily, preceded by recitations. On no account to exceed three lectures on one day, and the third to be a clinical lecture, occurring, say twice a week. Dissections to be continued during all suitable seasons, the time to be at the option of the student.

4. The spring and autumnal course to be dependent on each other. For instance, let Materia Medica, Chemistry, Anatomy, and Physiology, be taught in the spring; and Practice, Pathology, Surgery, Obstetrics, and Jurisprudence, in the fall.

Such is a rough sketch of a plan. Its advantages are manifest. The student would commence with the collateral sciences in the spring, and be fitted for the practical departments in the fall of the year. His time would be occupied sufficiently to make his studies a constant occupation, but not so much as to fatigue him beyond endurance, or deprive him of the necessary hours for reading and recreation. The smaller number of branches under consideration at one time, would prevent that strange confusion of ideas which is the misfortune of our present crowded winter courses. Finally, a repetitition of three such years would send out well-qualified practitioners, with their knowledge well arranged and practically useful.

To the teacher, other inducements will readily present themselves. It would require no more time than the present plan, while he could more easily adapt his hours to his business convenience, than in the crowd and hurry of the ordinary session, when he is necessarily compelled to yield much to the convenience of his colleagues. Pecuniarily, the number of students being equal and the fees being unchanged, he would receive the same emolument for the same labor.

The difficulties in the way will be found in the competition of money-seeking schools, which will cling to the old system and bid for students on the plea of less time and expense. But let the leading schools of New York and Philadelphia combine on such a plan, and they would soon crush out all opposition. It is hardly to be expected that the movement would be unanimous, but there is much reason to suppose that it would be finally successful. Another and more considerable opposition would arise from the students themselves, or that large class of them who are in search, not of an education, but a diploma. But the prestige attending graduation from such a school would do away with this. The argument is money; and those that have money (I speak here on the supposition that the expenses would be increased, simply by a prolonged city residence), will part with it for the eclat of a thorough education, or for the better motive of the education itself. Those that have no money get through now, with a system nearly as expensive, somehow; and I presume they would do so still. Let them run in debt, or carry along some temporary occupation. Such must oftener beget success than failure; and it is a peculiarity of poor but high-spirited students that they seek the best advantages.

But supposing that it very largely increased the expense of an education, what would be the general result? Men of energy and character find their way into the profession of law through greater difficulties than those with which we propose to environ the entrance to that of medicine. And others than men of energy and character had better stay out. I am aware that the tendency of the day is to cheapen all forms of education, to throw open every pathway to position and preferment. So far as this applies to that degree of education necessary to teach the poor man his rights and his duties to the body politic, all true men will go with this tendency; but this does not reach beyond a comprehensive and liberal system of common schools.

The further disposition to cheapen and make easy the pathway to the learned professions is a specious theory, which flatters only to destroy. It holds out inducements to the weak and indolent to assume the onerous responsibilities of a path of life beyond their abilities; it does them the greatest wrong that can by any possibility be done any man, by placing them in a false position, when a lifetime must be spent in vain regrets over the youthful vanity and folly which led them from the calm and peace of humbler duties to a struggle for which they are incompetent.

One of the greatest evils of our profession is the lack of recognition of its proper social position, and the low pecuniary estimate placed upon its services at the bedside. Two causes exist for this; one is the ease with which the profession is entered, the hordes which crowd into it attracted by its cheapness, and the competition for "custom," which naturally results. Another is the character of the men who make up its rank and file. It is of no use to say that the profession is not properly appreciated. Taken in the mass, it is. The verdict of Molière has been confirmed by the people, and the voice of an enlightened people is the voice of God, when through centuries it utters one unchanging opinion. The fault is in ourselves, and we alone can remedy it.

I suppose that a temporary result of the scheme here proposed would be a falling off in the number of students, but another and more important result would be an increased respect in the public mind for our art, and an increased emolument to the physician. Let it be carried out fully for a few years, and we should fill the profession with gentlemen, who would command social position, and elevate at once the character and the emolument of the physician. We should shut out the *profanum vulgus*, draw to us men of liberal minds, and place the American profession beyond that of any other nation in the prestige of its true nobility.

Finally, for I have occupied much space, all this can be done, but not at once; time for the preparation of the public mind is necessary, the schools should be brought to recognize the merits of the plan, and, above all, in our hot haste for reformation, we should beware lest we endanger our present grand old fabric.

The provision of partial summer courses, teaching some of the collateral departments only and conferring no degrees, at once suggests itself as a means, feasible at the present moment and likely to lead eventually to the broad reform I hope to live to see. In the meantime let the censure of the press and the profession fall heavily on all those inefficient schools which fail to meet the present standard of excellence.

To return to the comparison with which I started. We must remove one by one the timbers of our noble structure, replacing them cautiously by better materials; and in the end we shall altogether renovate it, giving it strength, solidity, and permanence; while, in the meantime, the rush of human life shall pass by it uninterrupted and unconscious of a change.

